# A randomized, multicenter and noninferiority study of amoxicillin plus berberine vs tetracycline plus furazolidone in quadruple therapy for *Helicobacter pylori* rescue treatment

**DOI:** 10.1111/1751-2980.12870

**Published:** 2020-06-09

**Authors:** Jian Zhang, Chuan Han, Wen Quan Lu, Na Wang, Si Ran Wu, Yong Xi Wang, Jin Ping Ma, Jie Hong Wang, Cheng Hao, Dong Hong Yuan, Na Liu, Yong Quan Shi

**Affiliations:** ^1^ State Key Laboratory of Cancer Biology, National Clinical Research Center for Digestive Diseases, Xijing Hospital of Digestive Diseases Air Force Military Medical University Xi'an Shaanxi Province China; ^2^ Department of Endocrinology General Hospital of the Western Theater Command Chengdu Sichuan Province China; ^3^ Department of Gastroenterology First Affiliated Hospital of Zhengzhou University Zhengzhou Henan Province China; ^4^ Department of Gastroenterology Xianyang Central Hospital Xianyang Shaanxi Province China; ^5^ Department of Gastroenterology Affiliated Hospital of Shaanxi University of Chinese Medicine Xianyang Shaanxi Province China; ^6^ Department of Gastroenterology Yan'an University Affiliated Hospital Yan'an Shaanxi Province China; ^7^ Department of Gastroenterology Second Affiliated Hospital of Xi'an Jiaotong University Xi'an Shaanxi Province China

**Keywords:** amoxicillin, berberine, *Helicobacter pylori*, rescue therapy

## Abstract

**Objective:**

*Helicobacter pylori* (*H. pylori*) infection is closely associated with gastric ulcers and gastric adenocarcinomas. We aimed to assess the efficacy and safety of a quadruple regimen with amoxicillin plus berberine vs tetracycline plus furazolidone in rescue therapy for *H. pylori* eradication.

**Methods:**

We conducted a randomized, open‐label, multicenter, noninferiority trial. Patients with previous treatment failures recruited from five centers were randomized (1:1) to receive a regimen with esomeprazole and bismuth plus either berberine and amoxicillin (the BA group) or tetracycline and furazolidone (the TF group) for 14 days. Their *H. pylori* infection status was confirmed 4‐8 weeks after treatment. The primary outcome was the eradication rate. The secondary outcomes included the rates of symptom improvement, compliance, and adverse events. This study was registered at ClinicalTrials.gov (NCT03609892).

**Results:**

Altogether 658 participants were consecutively enrolled. An intention‐to‐treat analysis demonstrated that the two regimens achieved a similar eradication rate (76.3% vs 77.5%; *P* = 0.781). The per‐protocol analysis reached a similar result (81.5% vs 85.0%; *P* = 0.278). The eradication rate reached in the BA group was greater than the pre‐established margin of noninferiority, at −10% (the lower bounds of the 95% CI were −7.66% and −9.43%, respectively). The rate of adverse events was lower for the BA group than the TF group (18.5% vs 26.1%, *P* = 0.024). Rates of compliance and symptom improvement were similar for the two therapies.

**Conclusion:**

The efficacy of both regimens in rescue treatment for *H. pylori* eradication was satisfactory, 14‐day BA‐based quadruple therapy is noninferior to the TF‐based therapy.

## 1 INTRODUCTION

It is generally accepted that *Helicobacter pylori* (*H. pylori*) infection plays a vital role in the development of chronic gastritis, gastric and duodenal ulcers, and gastric malignancy.^1^ Eradicating *H. pylori* in infected individuals help in the resolution of gastritis and upper gastrointestinal ulcers and may reduce the occurrence of gastric cancer.^2^, ^3^, ^4^ Thus, standard eradication therapy should be recommended to all patients with a known *H. pylori* infection.^5^ However, a continuous increase in bacterial antibiotic resistance is a huge challenge for *H. pylori* eradication. Unfortunately, the eradication efficacy of the classical standard triple therapy has been disappointing (being less than 80% for decades).^6^, ^7^ Resistance to the main antibiotics, such as clarithromycin and levofloxacin, in *H. pylori* eradication treatment has soared in recent decades.^8^ Previous studies have reported that clarithromycin resistance rates have soared to more than 30% in Japan and Italy, and more than 40% in Turkey, although those in Sweden and the Taiwan region remain relatively low (about 15%).^9^

*The Fifth Chinese national consensus report on the management of* H. pylori* infection* has recommended seven antibiotic combinations that include six kinds of antibiotics.^10^ Nevertheless, according to recent data from China, primary *H. pylori* resistance rates to the main antibiotics (clarithromycin, levofloxacin, and metronidazole) were 20%‐50%, 20%‐50% and 40%‐70%, respectively, which exceeded our expectations.^11^, ^12^, ^13^, ^14^, ^15^, ^16^, ^17^ Quadruple therapies comprising two kinds of antibiotics, such as furazolidone, tetracycline, and amoxicillin, along with proton pump inhibitors and bismuth, have been proven to reach a relatively satisfactory rate (> 90%) for those with high resistance to the other main antibiotics.^18^, ^19^ However, due to the high unavailability of furazolidone and tetracycline, researchers are searching for treatment regimens that are more effective and safer.

There have been several studies on the efficacy of traditional Chinese medicine for treating *H. pylori* infection. Some of them have found that certain kinds of herbs exert an excellent inhibitory effect on *H. pylori*.^20^, ^21^ As a traditional Chinese medicine, berberine is derived from herbs and is widely used to treat different digestive diseases, such as diarrhea and bacterial gastroenteritis.^22^ In addition, berberine is very inexpensive (0.2 Yuan [RMB]/tablet) in China and can easily be obtained over the counter. Recently, it has been reported that berberine can significantly lower the minimum inhibitory concentrations of tetracycline and amoxicillin against *H. pylori* strains, through the mechanism of decreasing the expression of *hefA* mRNA.^23^ Berberine has synergistic effects with some common antibiotics, suggesting that it could be useful when combined with other antibiotics for the eradication of antibiotic‐resistant *H. pylori* strains.^24^ Moreover, the inhibitory effect of berberine on *H. pylori* strains on patients with peptic ulcers has been tested in some pharmacological studies, showing that berberine can inhibit the activity of arylamine N‐acetyltransferase, thus inhibit the proliferation of *H. pylori*.^25^, ^26^, ^27^

We previously performed two prospective randomized controlled trials on the effect of berberine for *H. pylori* eradication. Our results verified that a berberine‐containing therapy (berberine, esomeprazole, amoxicillin and clarithromycin) was not inferior to the first‐line standard quadruple regimen (bismuth, esomeprazole, clarithromycin, and amoxicillin) in the local region (ClinicalTrials.gov ID: NCT02296021).^28^ We also reported that a bismuth‐containing therapy including berberine plus amoxicillin was not inferior to that including amoxicillin plus clarithromycin in the first‐line *H. pylori* eradication treatment (ClinicalTrials.gov ID: NCT02633930).^29^ These results support the idea that berberine may be effective against *H. pylori* resistance. To explore the clinical application of berberine, we conducted this multicenter, randomized, controlled, noninferiority study on the efficacy and safety of a brand‐new quadruple regimen including amoxicillin plus berberine in *H. pylori* rescue treatment for patients who had previously failed eradication therapy at least once.

## 2 PATIENTS AND METHODS

### 2.1 Study design

This multicenter, open‐label, randomized, prospective, noninferiority trial was carried out in outpatients at Xijing Hospital (Xi'an, Shaanxi Province, China), the Affiliated Hospital of Shaanxi University of Chinese Medicine (Xianyang, Shaanxi Province, China), Xianyang Central Hospital (Xianyang, Shaanxi Province, China), Yan'an University Affiliated Hospital (Yan'an, Shaanxi Province, China) and the Second Affiliated Hospital of Xi'an Jiaotong University (Xi'an, Shaanxi Province, China) from July 2018 to August 2019. The study was ratified by the Ethics Committee of each participating center. Written, informed consent was obtained from each participant before they were enrolled in the study. The principles of the CONSORT statement for randomized controlled studies were followed. We carried out the trial according to the Declaration of Helsinki 2013. This trial has been registered at the website of ClinicalTrials.gov (no. NCT03609892).

### 2.2 Participants

Participants infected with *H. pylori* in whom eradication treatment had failed at least once, such as standard triple and quadruple therapies with or without bismuth, were consecutively recruited for our study. Eligibility criteria were as follows: (a) adult participants aged between 18‐70 years and of either sex; (b) patients in whom *H. pylori* eradication therapy had failed during the previous 2 years and had discontinued eradication therapy for more than 2 months; and (c) women who were not pregnant or lactating (child‐bearing women were asked to use a medically acceptable contraception during the study period and the subsequent 30 days after the study. Exclusion criteria were: (a) patients who had previously used tetracycline‐ and furazolidone‐based antibiotics to eradicate *H. pylori* infection; (b) contraindications to the study drugs; (c) with substantial organ impairment or severe or unstable cardiopulmonary or endocrine diseases; (d) constantly use of antiulcer drugs, including proton pump inhibitors within 2 weeks before the ^13^C/^14^C‐urea breath test (UBT), antibiotics or bismuth complexes for more than thrice within the month before screening; (e) pregnant or lactating; (f) a history of upper gastrointestinal surgery; (g) Barrett esophagus or highly atypical hyperplasia and symptoms of dysphagia; (h) evidence of bleeding or iron deficiency anemia; (i) previous history of malignancy; (j) a history of drug or alcohol abuse in the previous year; (k) systemically used corticosteroids, nonsteroidal anti‐inflammatory drugs, anticoagulants, or platelet aggregation inhibitors (except for using aspirin at less than 100 mg/d); (l) had been enrolled in another clinical trial in the previous 3 months; (m) psychological problems or showed poor compliance; or (n) refused to participate or provide their written informed consent.

The eligible patients in each center were randomly allocated (1:1) to take either of the two therapies for 14 days: (a) the berberine and amoxycillin (BA)‐based quardruple therapy group, a regimen including berberine 300 mg thrice daily (Yunnan Mingjinghengli Pharmaceutical, Kunming, Yunnan Province, China), amoxycillin 1 g twice daily (Zhuhai United Laboratories, Zhuhai, Guangdong Province, China), esomeprazole 20 mg twice daily (AstraZeneca, Södertälje, Sweden) and colloidal bismuth pectin 200 mg twice daily (Shanghai Diran Dancheng Pharmaceutical, Zhengzhou, Henan Province, China); or (b) the tetracycline and furazolidone (TF)‐based quardruple group, a regimen including tetracycline 500 mg thrice daily (Guangdong Huanan Pharmaceutical, Dongguan, Guangdong Province, China), furazolidone 100 mg twice daily (Yunpeng Pharmaceutical, Linfen, Shanxi Province, China), esomeprazol 20 mg twice daily (AstraZeneca), and colloidal bismuth pectin 200 mg twice daily (Shanghai Diran Dancheng Pharmaceutical). There was an independent research assistant at each center and the independent random number sequences used at each center were generated by a third party.

### 2.3 *H. pylori* infection detection

Detection of *H. pylori* infection was performed by using the ^13^C/^14^C‐UBT, *H. pylori* stool antigen test (HpSAT), histological confirmation of *H. pylori*, or the *H. pylori* rapid urease test (RUT). *H. pylori* infection was confirmed when the result of at least one of the abovementioned tests was positive.

The participants were assessed for *H. pylori* infection by either the ^13^C/^14^C‐UBT or HpSAT (for participants with a positive RUT result or a histological confirmation of *H. pylori* but negative UBT results before treatment) at 4 to 8 weeks after the completion of therapy.

### 2.4 Procedure

The participants were randomly allocated to receive a 14‐day BA‐based or TF‐based quadruple regimen. Each participant's characteristics and medical history were obtained at enrollment. We also collected data on the occurrence of their clinical symptoms, including vomiting, nausea, abdominal pain, flatulence, diarrhea, constipation, early satiety, belching, hiccup, dysgeusia, headache, dizziness, drowsiness, fever, acid reflux, and heartburn. The severity of symptoms was categorized from 0 to 3 (0, 1, 2, and 3, represent none, mild, moderate, and severe, respectively). The frequency of symptoms was also categorized from 0 to 3 (0, 1, 2, and 3, represents none, mild, moderate, and severe, respectively). A total score of each symptom was calculated as follows: total score = frequency score × severity score.

**FIGURE 1:**
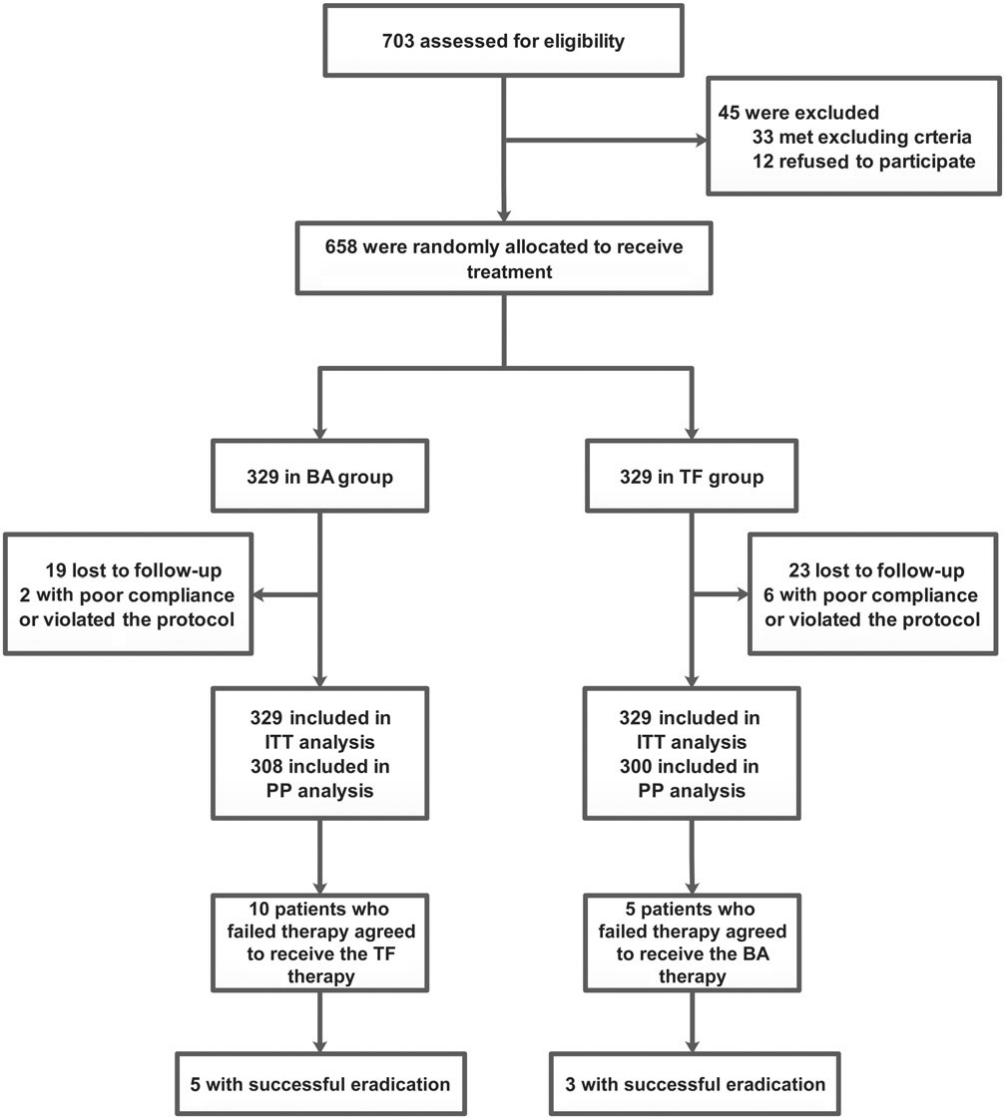
Flow diagram of the study. Abbreviations: BA, berberine and amoxicillin; ITT, intention‐to‐treat; PP, per‐protocol; TF, tetracycline and furazolidone

The participants were required to come back to the hospitals after they had completed treatment for an assessment of their compliance, adverse events and symptom improvement. The participants were informed of the common adverse events and were instructed to record these events on a predesigned case report form. A four‐point scale, which included none, mild (bothersome discomfort that did not interfere with normal life), moderate (sufficient discomfort to cause interference with normal life), and severe events (discomfort potentially meriting discontinuation of the therapy), was designed to evaluate adverse events prospectively. Participants were asked to stop using proton pump inhibitors and histamine‐2 receptor antagonists for at least 14 days before any examination. The technicians carrying out the *H. pylori* tests were blinded to the therapies the participants received. The whole flow diagram of the study is shown in Figure 1 **.**

### 2.5 Outcomes

The primary outcome was the efficacy of *H. pylori* eradication treatment, as evaluated at least 4 weeks after the completion of the therapy, and the secondary outcomes included the overall rates of adverse events, improvement in clinical symptom (defined as improved when the total clinical symptom score was reduced by ≥ 50%, as evaluated at the 2nd week of the treatment and at 4 weeks after completion of the therapy) and participants' compliance. Patients who took less than 80% of all medications were determined to have a poor compliance after counting unused medications at completion.

### 2.6 Statistical analysis and calculation of sample size

The efficacy of the quadruple regimen using tetracycline and furazolidone for *H. pylori* eradication has been reported to range from 75% to 90% for *H. pylori* rescue therapy.^30^, ^31^, ^32^ We assumed we wouldachieve an 80% eradication rate in both groups, a noninferiority margin of δ = −10% with a β power of 90% and an α of 0.05 (one‐sided). Based on the *H. pylori* eradication rate we assumed, the per treatment regimen required at least 274 participants to indicate noninferiority between the two groups. A 20% loss to follow‐up rate was taken into consideration, and we expected to recruit at least 329 participants for each group (658 participants in total) for this trial. BA therapy was considered noninferior to TF therapy if the lower bound of the 95% confidence interval (CI) was higher than −10%; otherwise, noninferiority was not concluded. *P* value of less than 0.05 was considered statistically significant.

## 3 RESULTS

### 3.1 Baseline characteristics of the participants

From July 2018 to August 2019, 703 patients were evaluated for eligibility; in all, 658 were enrolled in the trial and randomized to receive the 14‐day BA‐based or TF‐based quadruple regimen. In total, 19 participants in the BA group and 23 in the TF group were lost to follow‐up (we lost contact with all 42 participants and none of them finished the study). Additionally, two participants (one failed to finish the tests after treatment completion and the other discontinued treatment without authorization) in the BA group and six participants (three failed to finish the tests after the completion of treatment, two discontinued treatment without authorization, and the other was accidentally pregnant) in the TF group violated the study protocol or showed poor compliance. We excluded all 50 of these participants from the pre‐protocol (PP) analysis and regarded them as eradication failures in the intention‐to‐treat (ITT) analysis. As shown in Table 1, there were no statistical differences in patients’ baseline data, clinical characteristics or the antibiotics used in previous treatment regimens between the two groups. Moreover, nine participants had a positive RUT result or histological confirmation before treatment, and we obtained their endoscopic and pathological results on enrollment (two participants were diagnosed with ulcers, five with chronic atrophic gastritis, and the other two with chronic atrophic gastritis with intestinal metaplasia).

**TABLE 1:** Baseline characteristics of the participants

	BA group (N = 329)	TF group (N = 329)	*P* value
Age, y (mean ± SD)	48.84 ± 12.27	46.72 ± 12.90	0.108
Sex, n (male/female)	160/169	160/169	1
Body mass index, kg/m^2^ (mean ± SD)	22.74 ± 2.88	22.36 ± 3.14	0.111
Ethnicity, n (%)			
Han	325 (98.8)	327 (99.4)	0.686
Others	4 (1.2)	2 (0.6)	
Number of previous eradication attempts, n (%)			
1	297 (90.3)	301 (91.5)	0.297
≥2	32 (9.7)	28 (8.5)	
Comorbidities, n (%)			
Diabetes	13 (4.0)	10 (3.0)	0.620
Hypertension	14 (4.3)	17 (5.2)	
Others	5 (1.5)	7 (2.1)	
Previous antibiotic therapies (person‐time)			0.322
Amoxicillin + clarithromycin	275	266	
Tinidazole + clarithromycin	59	72	
Metronidazole + amoxicillin	32	24	
Metronidazole + clarithromycin	6	3	
Metronidazole + levofloxacin	1	0	

*Abbreviations*: BA, berberine and amoxicillin; TF, tetracycline and furazolidone; SD, standard deviation.

### 3.2 Efficacy of *H. pylori* eradication therapy

A total of 603 of the 608 participants who completed the study underwent a ^13^C/^14^C‐UBT, and only five of nine participants with a positive RUT result and histological confirmation of *H. pylori* required an HpSAT of the posttreatment status of *H. pylori* infection. As indicated in Table 2, according to the ITT analysis *H. pylori* was successfully eradicated in 76.3% of patients in the BA group and in 77.5% of those in the TF group. On PP analysis, the eradication rate reached 81.5% and 85.0% in the BA and TF groups, respectively. There was no statistical difference between the two regimens (*P* = 0.781 and 0.278 for the ITT and PP analysis, respectively). Moreover, the lower bound of the 95% CI for the eradication rate difference in the BA therapy was greater than the pre‐established marginal value of noninferiority of −10% (−7.66% and − 9.43%, respectively).

**TABLE 2:** *Helicobacter pylori* eradication rates

	ITT analysis		PP analysis	
	BA group	TF group	BA group	TF group
n/N (%)	251/329 (76.3)	255/329 (77.5)	251/308 (81.5)	255/300 (85.0)
95% CI	71.7‐80.9	73.0‐82.0	77.1‐85.9	80.9‐89.1
RD 95% CI	−7.66, 5.22		−9.43, 2.42	
*P* value	0.781		0.278	

*Abbreviations*: BA, berberine and amoxicillin; CI, confidence interval; ITT, intention‐to treat; PP, per‐protocol; RD, rate difference; TF, tetracycline and furazolidone.

### 3.3 Rates of adverse events, compliance, and symptom improvement

As shown in Table 3, 61 (18.5%) participants in the BA group and 86 (26.1%) participants in the TF group experienced adverse events (except dark stool that was related to the use of bismuth) when receiving treatment. The overall frequency of adverse events was found to be significantly lower in the BA group than in the TF group (*P* = 0.024). The severe adverse events rates were comparable in the two groups (*P* = 0.722). The main adverse events included taste distortion, nausea, vomiting, abdominal pain, bloating, diarrhea, dizziness, headache, skin rash, and fatigue (Table 4). All patients had recovered from the adverse events 1‐2 weeks after the therapy ceased. Three participants in the BA group and four in the TF group stopped the therapy owing to the severe adverse events. The two therapeutic regimens showed similar compliance rate and symptom improvement rate at the 2nd and 6th weeks.

**TABLE 3:** Rates of adverse events, compliance and symptom improvement (n/N, %)

	BA group	TF group	*P* value
Overall adverse events	61/329 (18.5)	86/329 (26.1)	0.024
Severe adverse events	3/329 (0.9)	4/329 (1.2)	1.000
Compliance rate	305/329 (92.7)	296/329 (90.0)	0.267
2‐week symptom improvement	196/283 (69.3)	166/266 (62.4)	0.105
6‐week symptom improvement	239/283 (84.5)	216/266 (81.2)	0.365

*Abbreviations*: BA, berberine and amoxicillin; TF, tetracycline and furazolidone.

**TABLE 4:** Frequency of adverse events in the two groups (n/N, %)

	BA group	TF group	*P* value
Taste distortion	10/61 (16.4)	17/86 (19.8)	0.670
Nausea	18/61 (29.5)	29/86 (33.7)	0.720
Abdominal pain	13/61 (21.3)	20/86 (23.3)	0.842
Vomiting	11/61 (18.0)	15/86 (17.4)	1.000
Bloating	12/61 (19.7)	8/86 (9.3)	0.089
Diarrhea	14/61 (23.0)	16/86 (18.6)	0.539
Dizziness	2/61 (3.3)	6/86 (7.0)	0.470
Headache	3/61 (4.9)	2/86 (2.3)	0.649
Skin rash	1/61 (1.6)	2/86 (2.3)	1.000
Fatigue	6/61 (9.8)	9/86 (10.5)	0.565

*Abbreviations*: BA, berberine and amoxicillin; TF, tetracycline and furazolidone.

## 4 DISCUSSION

Due to a rapid increase in antibiotic resistance worldwide, the overall global eradication rate of *H. pylori* using the standard triple regimen has dropped to less than 80%.^33^ While genetic polymorphisms in *CYP2C19*, unsatisfactory compliance, and the bacterium itself are considered the reasons for this phenomenon, antibiotic resistance is regarded as the most important factor, especially in China.^17^ A study in Shanghai reported that *H. pylori* resistance rose from 8.6% to 20.7% for clarithromycin and from 10.3% to 32.5% for levofloxacin from 2000 to 2009. However, all strains remained unresistant to furazolidone and amoxicillin, and only one strain of the bacterium was resistant to tetracycline.^34^ Tetracycline and furazolidone‐based quadruple therapies have also achieved a satisfactory eradication rate in China. Some authors have reported that a quadruple regimen containing tetracycline attained an eradication rate of 89.4% on ITT analysis and 91.6% on PP analysis.^35^ However, due to adverse events and the lack of their availability, there is a great limitation in the use of tetracycline and furazolidone in the treatment of *H. pylori* infection.

Berberine is isolated from *Coptis chinensis* and is known for its antiprotozoal, antibacterial, and anti‐inflammatory abilities. Studies have shown that berberine may effectively inhibit the growth of *H. pylori*, with a zone of inhibition reaching 25 mm in diameter.^36^ A series of trials have demonstrated that berberine has several antibacterial mechanisms. Berberine use can lead to DNA damage in bacteria.^37^, ^38^, ^39^ The inhibition of FtsZ, the cell division protein, was found to be the main mechanism of action of berberine against bacteria.^40^ Studies have shown that berberine can effectively treat infections with drug‐resistant bacteria by exerting synergistic effects with different antibiotics.^41^ Moreover, studies have demonstrated that berberine can modulate the cytokines in cells and decrease the level of free radicals in the mucous membrane. Moreover, high concentrations of berberine can be absorbed rapidly in the gastric mucosa with long‐lasting effects.^42^ Berberine has also been found to alleviate antibiotic‐related diarrhea by modulating the intestinal microbiome.^43^ Furthermore, berberine has been proven to relieve diarrhea by inhibiting the contraction of intestinal smooth muscle cells.^44^ A meta‐analysis has also demonstrated that berberine‐containing therapies will enhance the efficacy and decrease the incidence of adverse effects.^45^

To attain high‐level clinical evidence, we conducted this large, multicenter, prospective, randomized, controlled, noninferiority study. The results are encouraging and demonstrate that quadruple therapy containing berberine and amoxicillin reached an eradication rate similar to that of a classical therapy consisting of tetracycline plus furazolidone. The approximately 80% eradication rate in our study is similar to the findings of another study in China.^46^

Several factors may be responsible for the results. First, our patients were mainly from northwest China, which has a different population from those that studied in previous trials showing high efficacy. Moreover, a previous study has shown increasing resistance to different antibiotics, including tetracycline, in northwest China.^47^ Second, another study has demonstrated a new drug‐resistant gene (*rfaF*) that is responsible for cross‐resistance to tetracycline and other antibiotics.^48^ A Chinese study reported that 63.1% (736/1167) of strains from patients who experienced eradication failure had secondary resistance to furazolidone.^49^ Third, different antimicrobial susceptibility appeared in *H. pylori* strains isolated from different areas, which may make it difficult to detect and thus influence the efficacy of the treatment.^50^

Adverse events and participants’ compliance were associated with the efficacy of *H. pylori* treatment. The two groups in this study showed comparable and satisfactory compliance rates (both > 90%), which might be attributed to our close follow‐up. The BA group had a significantly lower overall frequency of adverse events (*P* = 0.024), which is better for patient compliance, especially moderate and severe adverse events that may be responsible for treatment cessation. Some participants had reported that berberine‐containing medication reduced diarrheal symptoms, which can be caused by high‐dose antibiotics. Regarding symptom improvement, we found that in more than 60% and more than 80% of patients the symptoms significantly improved at 2 and 6 weeks after the initiation of the treatment, respectively. Although the two regimens did not show any significant statistical difference, berberine and amoxicillin showed relatively higher rates of symptom improvement compared with tetracycline and furazolidone. Three of five participants in the TF group failed to achieve *H. pylori* eradication and received the BA regimen, achieving successful eradication 2‐3 months later. These findings indicate that BA‐based quadruple therapy has the potential to overcome resistance to tetracycline or furazolidone. More importantly, most of our participants (>90%) had used amoxicillin‐containing therapy in previous eradication regimens, which may indicate that berberine is capable of overcoming resistance to amoxicillin or even of eradicating *H. pylori* without the use of other antibiotics.

Previous studies have indicated that the number of treatments and previous therapies may influence the efficacy of rescue eradication therapy. In our trials the previous therapies and numbers of treatment were comparable in both groups.

There were some limitations to our study. First, our centers were all located in northwest China, and most patients were from the area surrounding Shaanxi Province. Whether the berberine plus amoxicillin regimen is available in other countries or other areas in China needs to be confirmed. Second, the dosage of berberine was determined based on our experience; whether higher doses of berberine can improve efficacy requires further research. Moreover, a series of assessments of the factors affecting the eradication efficacy of the two regimens, such as polymorphisms of CYP2C19 virulence factors are needed. In future studies we will assess the factors that may help us select the best antibiotics and proton pump inhibitors. In addition, we did not complete *H. pylori* culture, which may result in difficulties in analyzing whether susceptibility and drug resistance of *H. pylori* strains were balanced between the groups. Therefore, we intend to culture *H. pylori* strains from participants to analyze the characteristics of *H. pylori* strains in further trials.

## 5 CONCLUSIONS

In conclusion, to our knowledge, our study is the first to provide an innovative approach to the rescue therapy for *H. pylori* eradication involving the addition of a traditional Chinese medicine. Based on our results, the efficacies of these two regimens were satisfactory, and berberine plus amoxicillin quadruple therapy was noninferior to tetracycline plus furazolidone quadruple therapy.

## CONFLICT OF INTEREST

None.
